# Associations of the perceived and objective neighborhood environment with physical activity and sedentary time in New Zealand adolescents

**DOI:** 10.1186/s12966-017-0597-5

**Published:** 2017-10-25

**Authors:** Erica Hinckson, Ester Cerin, Suzanne Mavoa, Melody Smith, Hannah Badland, Tom Stewart, Scott Duncan, Grant Schofield

**Affiliations:** 1 Auckland University of Technology, Human Potential Centre, Auckland, New Zealand; 2 Institute for Health and Ageing, Australian Catholic University, Melbourne, Australia; 3 School of Public Health, The University of Hong Kong, Hong Kong, China; 4 Melbourne School of Population and Global Health, The University of Melbourne, Melbourne, Australia; 5 SHORE & Whariki Research Centre, Massey University, Auckland, New Zealand; 6 School of Nursing, The University of Auckland, Auckland, New Zealand; 7 Centre for Urban Research, RMIT University, Melbourne, Australia; 8 Auckland University of Technology, Centre for Child Health Research, Institute of Public and Mental Health Research, Private Bag 92006, Auckland, New Zealand

**Keywords:** Accelerometer, Activity friendly, Urban environment, Youth

## Abstract

**Background:**

There is accumulating evidence supporting the association between neighborhood built environments and adults’ physical activity (PA) and sedentary time (ST); however, few studies have investigated these associations in adolescents. A better understanding of the features of the built environment that encourage PA or ST is therefore of critical importance to promote health and wellbeing in adolescents. The aim of this study was to estimate the associations of GIS-determined and perceived walkability components in individual residential buffer zones with accelerometer-assessed moderate-to-vigorous physical activity (MVPA) and ST in adolescents.

**Methods:**

The Built Environment in Adolescent New Zealanders (BEANZ) study was conducted in two cities (Auckland and Wellington) during the 2013-2014 academic school years. The exposure measures were subjective and objective environmental indices of activity-friendliness using four residential buffers. Road network buffers were calculated around participant’s residential addresses using the sausage buffer approach at 250 m, 500 m, 1 km, and 2 km scales. A 25 m radius was used for the buffers. Data were analysed using Generalized Additive Mixed Models in R.

**Results:**

Data were analysed from 524 participants (15.78 ± 1.62 years; 45% male). Participants accumulated ~114 min/day of moderate-to-vigorous PA (MVPA) and ~354 min/day of ST during accelerometer wear-time (~828 min/day). The estimated difference in MVPA between participants with the 1st and 3rd quartiles observed values on the composite subjective environmental index of activity-friendliness (perceived land use mix - diversity, street connectivity and aesthetics) was equivalent to ~8 min/day (~56 MVPA min/week) and for the *objective environmental index of activity-friendliness* (gross residential density and number of parks within 2 km distance from home) was ~6 min of MVPA/day (~45 MVPA min/week). When both indices were entered in a main-effect model, both indices remained significantly correlated with MVPA with sex as a moderator. The predicted difference in sedentary time between those with the minimum and maximum observed values on the *subjective index of non-sedentariness* was ~20 min/day.

**Conclusions:**

The combined assessment of the main effects of subjective and objective indices of activity-friendliness on NZ adolescents’ PA and ST showed positive relationships with MVPA for the subjective index only. The subjective index was a significant correlate of PA in both girls and boys, while the objective index was significant only in boys when sex was entered as a moderator. Further research is warranted to understand the relationships of ST with the built environment.

## Background

Evidence suggests that low physical activity (PA) and prevalence of sedentary behaviors in adolescence are negatively associated with adolescent health outcomes such as obesity, cardiovascular illnesses and metabolic disorders [1]. Hancox, Milne & Poulton [2] reported that sedentary behaviors such as television viewing were associated with obesity, poor fitness, smoking, and hypercholesterolaemia in adulthood [2]. Globally, PA in adolescents is declining and sedentary time (ST) is increasing [3, 4]. The recent Global Report Cards on PA has awarded a D grade on both overall PA and ST around the world for children and youth [4]. While New Zealand ranked second of 38 countries on the Global Report Cards for PA, approximately one-third of children and youth were insufficiently active for health, and that adolescents accumulated less PA, less active transport, and more sedentary behavior than their younger peers [5].

To date, most efforts to improve PA and ST have focused on changing individual behaviors, with varying degrees of effectiveness. A meta-analysis of 358 studies involving over 99,000 healthy adults showed that behavioral approaches were only moderately effective, with a mean effect size that equated to 14.7 min difference in PA per week between treatment and control groups [6]. When considering the public health implications of these findings, it is also important to consider that individually-based behavioral interventions: (1) are only offered to a small portion of the population (study participants) and so have limited reach, (2) affect change in a sub-sample of the study population only, and (3) have long-term effects in an even smaller subsample of the study population. Indeed, using the RE-AIM framework (reach, efficacy, adoption, implementation, maintenance [7]) it is clear that behavioral interventions are significantly limited in their ability to cause sustained and meaningful levels of behavioral change across a large portion of the population.

A socio-ecological approach suggests that the wider environment and contexts in which PA and ST take place should be considered in efforts to encourage behavior change and support healthy behaviors; therefore, the built environment plays an important role in the formation of habits and health behavior [8]. Recent evidence suggests a link between PA and the built environment in youth [9], however, it is not clear if an equivalent relationship exists with ST. Certain features of the built environment may discourage engagement in outdoor activities, and therefore encourage participation in indoor sedentary activities such as videogames [10]. Nonetheless, there is currently limited evidence that explores the nature and magnitude of these relationships. Most of the work in this age group has focused on self-reported television (TV) viewing as a proxy for sedentary behavior [11–13].

An advance to self-reported measures of ST and PA is the use of accelerometry. Objective measures have some component of measurement error; however, there is no evidence that any systematic bias, such as under estimation, introduces bias in the association between the environment and the behavior. The two studies [14, 15] that examined objectively measured ST and PA (using Actigraph accelerometers) with built environment measures (through GIS) found boys were less sedentary when living in neighborhoods with parks and residences with cul-de-sac networks compared with those living in neighborhoods with highest residential density, the lowest vacant acreage, and the most recreational and school facilities [14]. Jago and colleagues reported that only sidewalk characteristics were positively associated with PA and negatively associated with ST in male youth (females were not investigated) [15].

It has been shown that environmental perceptions are stronger correlates of activity among adolescents than objective measures [16]. Particular perceived environmental features may relate better to PA because of the familiarity of the neighborhood among active residents. While objective measures reduce the subjectivity that is associated with perceived measures, they may not capture accurately the relationship that exists between residents’ PA and the environment [17]. McGinn et al. [18] reported an overall poor level of agreement between objective and perceived measures of the built environment in adults. However, when both objective and perceived measures were combined in the same model independent associations were observed with PA. This suggests the necessity to include both objective and perceived measures of the built environment when examining the relationship between the built environment and PA [18].

The aim of this study was to estimate the associations of GIS-determined and perceived walkability components in individual residential buffer zones with accelerometer-assessed MVPA and ST in adolescents by providing (for the first time) a *combined* assessment of the contribution of subjective and objective indices of activity-friendliness to the explanation of adolescents’ PA and ST.

## Methods

### Study design

The Built Environment in Adolescent New Zealanders (BEANZ) study is a cross-sectional investigation of the associations among measures of the built environment and PA and sedentary behavior in New Zealand adolescents [19]. Data were collected during the 2013-2014 academic school years from participants in school settings during school hours. Ethical approval was received by the Host Institution’s ethics committee.

### Participant recruitment

Participants aged (12–18 years) were recruited from eight high schools across two of New Zealand’s largest cities, Auckland and Wellington (total resident population 1,415,550 and 190,956, comprising 33 and 5% of the total New Zealand population respectively [20]). In order to address our aim, we needed to maximise built environment and socioeconomic heterogeneity in the sample, therefore purposefully selected the eight high schools. Using a GIS-derived walkability index (street connectivity, residential density and land use mix [21]) and deprivation data (NZ dep 2006 [22]) as an indicator of socio-economic status (SES), Auckland and Wellington urban meshblocks (smallest census tract units available in New Zealand) were classified into four strata: higher walkable, higher SES; higher walkable, lower SES; lower walkable, higher SES and lower walkable, lower SES. The walkability index was based on an index developed in the US [21] and was created as part of an international study that used standardised GIS measures [23]. Components of this walkability index have been associated with self-report and objectively measured physical activity in New Zealand adults [24].

Meshblocks with the top four walkability/SES deciles were classified as higher walkable/SES, and meshblocks with the bottom four walkability/SES deciles were classified as lower walkable/SES. To maximise variability, meshblocks with walkability/SES in the middle deciles, 5 and 6, were excluded. A similar sampling strategy was used in our previous study of the environmental correlates of PA in adults; the heterogeneity generated by this technique permitted several meaningful associations to be detected with residents’ activity in adults [24, 25] and children [26]. Our aim was to recruit participants living in each of the four strata described below. Therefore, we recruited students from eight schools (six in Auckland and two in Wellington). We chose to recruit from eight schools in order to balance recruitment costs, likely sample size, and location diversity. The eight schools were selected partly based on location near meshblocks in each of the four strata, and partly based on convenience. The assessment of the school’s location near meshblocks was undertaken by visually assessing the number of meshblocks in each strata within 1600 m of the school. Only one school we approached declined to participate. Subsequent school selection was based on the strata that needed to be balanced. For example, if our sample was lacking students residing in high walkability, low income meshblocks, then we tried to ensure that the next school was located near such meshblocks. In some cases we selected schools because they had more than one strata within 1600 m. Within each school, all pupils were invited to participate, regardless of the stratum in which they resided. Participation required written, informed consent from a parent or caregiver and informed written assent from the adolescent.

### Measures

#### Objectively-assessed physical activity and sedentary time

The GT3X+ Actigraph accelerometer was used to objectively estimate minutes of PA and ST over a 7-day period. The GT3X+ is a small, sturdy and water-resistant device that records the intensity, rate, and duration of activity. Reliability and validity properties of this device have been documented extensively [27–30]. Participants were asked to wear the accelerometer above the right hip during waking hours and to remove it only for water activities (e.g. swimming, bathing). Raw data were downloaded in Actilife 6 (Actigraph, Pensacola, FL) and aggregated to 30-s epochs with the low frequency extension filter enabled. This was done to increase the sensitivity to movement and maintain comparative ability with older models [31]. The count thresholds established by Evenson and colleagues [32] were used to assess activity intensity (i.e., MVPA >2295 counts per minute [CPM]; sedentary <100 CPM). The average minutes per day of MVPA and sedentary time were treated as outcome measures. Data were then exported to MeterPlus (Santech, San Diego, CA) where each participant’s data were visually inspected and assessed for wear time. Non-wear time was defined as at least 60 min of consecutive zero counts [33]. A valid day was defined as at least 10 h of wear time during weekdays and at least 8 h during weekend days [34]. Participants were included in the final analysis if they had at least 5 valid days of data, including at least one weekend day [31].

#### GIS measures of the built environment

GIS data were used to objectively characterise the built environment surrounding the primary home address of each participant and were applied across a range of road network buffers in order to evaluate differences between various limits of exposure.

Road network buffers were calculated around participant’s residential addresses using the sausage buffer approach [35] at 250 m, 500 m, 1 km, and 2 km scales. A 25 m radius was used for the buffers. Road network data was sourced from Core Logic Ltd. (2015) and a walkable road network was created by removing roads with no pedestrian access (e.g. motorways) prior to analyses.

Six measures of the physical environment were calculated for each buffer in GIS. *Gross residential density, Street intersection density, Cul-de-sac density, Transit stop density, Number of parks, Land use mix* (please see Appendix 1 for more detail). These measures were subsequently used to derive a composite objective index of activity-friendliness (see Data analyses section).

##### Neighborhood environment Walkability scale – Youth (NEWS-Y)

The NEWS-Y assesses perceived neighborhood attributes related to walking, PA, and sedentary behavior [36, 37]. The original NEWS-Y consists of eight subscales: residential density, land use mix – diversity, land use mix – access, street connectivity, walking facilities, aesthetics, pedestrian/automobile traffic safety, and crime. However, subsequent psychometric work on the adult version of the NEWS [31, 38], in addition to a confirmatory factor analysis of the data collected in this study, indicated that the 6-item land use mix – access subscale should be split into a 3-item land use mix – access scale and three single-item scales, being: parking difficulty in local shopping areas (*Parking is difficult in local shopping areas*); physical barriers to walking (*There are major barriers to walking (alone or with someone) in my local area that make it hard to get from place to place (for example, freeways, railway lines, rivers)*); and hilly streets (*The streets in my neighborhood are hilly, making my neighborhood difficult to walk in (alone or with someone)*). Hence, the version of the NEWS-Y used in the current study consisted of 11 subscales (seven original, one reduced, and three single items, see Appendix 2 for more detail).

#### Data analyses

Descriptive statistics were computed for the whole sample with at least five valid days of accelerometer data (including at least one weekend day). Associations of perceived environmental attributes with objectively were determined using additive mixed models (GAMMs; [39, 40]). GAMMs can model data with various distributional assumptions, account for dependency in error terms due to clustering (participants recruited from selected schools and neighborhoods), and estimate complex, dose-response relationships of unknown form [40]. Preliminary analyses based of residuals and model fit (Akaike’s information criterion, AIC) indicated that GAMMs with Gamma variance and logarithmic link functions would be most appropriate to models objectively-measured MVPA and sedentary time.

Main-effect GAMMs estimated the dose-response relationships of each perceived and objective environmental attribute with objectively-measured MVPA and sedentary time. All models were adjusted for city (Auckland or Wellington), socio-demographic covariates (parental marital status, highest educational attainment in household, length of residence in the neighborhood, child’s sex, age and race/ethnicity), accelerometer wear time, and administrative-unit-level socio-economic status (defined as the median household income of the administrative unit of residence) (hereafter named ‘covariates’). Length of residence at the current address was not included as a covariate because it was highly correlated with length of residence in the neighborhood. The latter was deemed to be more important in capturing familiarity with the place of residence and hence was selected for inclusion in the list of covariates. For the purpose of the analyses, several socio-demographic covariates were dichotomized given the low frequencies of some of the original covariate categories. Specifically, race/ethnicity was dichotomized as ‘European origins’ (New Zealand Europeans) and ‘non-European origins’ (Maori, Pacific, Asian and other). Educational attainment was dichotomized as ‘less than tertiary education’ and ‘completed tertiary education’. Marital status was categorized into ‘living with a partner’ and ‘not living with a partner’.

We estimated separate covariate-adjusted GAMMs for each environmental attribute given that several attributes were substantially correlated. Perceived and objective environmental attributes that were associated with MVPA were used to construct, respectively, subjective and objective environmental indices of activity friendliness, while those associated with sedentary time were used to construct subjective and objective environmental indices of non-sedentariness. Subjective and objective measures were not combined into one index because they represent different constructs and there is evidence that the level of correspondence between them is, at best, moderate [41, 42]. Associations between objectively-assessed environmental attributes and MVPA/ST are the most appropriate type of information for guiding environmental interventions (aspects of the environment that need to be modified), while associations between perceptions of the environment and MVPA/ST are best at informing interventions aimed at changing residents perceptions of their neighbourhood through environmental changes or behavioral strategies.

The environmental indices of activity-friendliness were computed by summing up the standardized scores (z-scores) of the variables that were positively related and subtracting the standardized scores of variables that were negatively related to MVPA. The environmental indices of non-sedentariness were computed by summing up the standardized scores (z-scores) of the variables that were negatively related and subtracting the standardized scores of variables that were positively related to sedentary time. Separate GAMMs with each of the relevant indices of activity-friendliness and non-sedentariness as predictors were estimated. We also estimated GAMMs with both relevant perceived and objective indices simultaneously entered as predictors.

Curvilinear relationships of environmental attributes with MVPA and sedentary time were estimated using non-parametric smooth terms in GAMMs, which were modelled using thin-plate splines [40]. Smooth terms failing to provide sufficient evidence of a curvilinear relationship (based on AIC) were replaced by simpler linear terms. Separate GAMMs were run to estimate environmental attribute by sex interaction effects. The significance of interaction effects was evaluated by comparing AIC values of models with and without a specific interaction term. An interaction effect was deemed significant if it yielded a > 5-unit smaller AIC than the main effect model [43, 44]. Significant interaction effects were probed by computing sex-specific association.

There were 9.5% of cases with missing data on at least one of the variables. The presence of missing data on specific variables was related to other variables included in the study, i.e., data were at least missing at random [45]. Consequently, ten imputed datasets were created for the main regression analyses as recommended by Rubin [45] and van Buuren [46]. Analyses based on complete data only when missing data are MAR can yield biased results, while analyses based on properly-conducted multiple imputations do not [45]. Multiple imputations were performed using chained equations (MICE; [46]) accounting for administrative-unit-level and school cluster effects arising from the sampling strategy employed in each study site. The ten imputed datasets were created in R (R Development Core Team, 2014) using the package ‘MICE’ and following the model-building and diagnostic procedures outlined by van Buuren [46]. All analyses were conducted in R (R Development Core Team, 2014) using the packages ‘car’ [47] and ‘mgcv’ [40].

## Results

Of the 5883 pupils invited, 752 adolescents participated in this study (13% response rate). An average of 94.1 (SD = 31.2) participants provided consent from each school (range = 39–142). Participants were excluded if they did not wear the accelerometer or did not have sufficient accelerometer data (*n* = 150), did not have address data (*n* = 23), or did not complete the survey (*n* = 55), yielding a final sample of 524 participants (44.5% male) aged 15.8 (1.6) years. When compared with participants who did not wear an accelerometer or had fewer than five valid days of accelerometer data, those who had at least five valid days of wearing time were more likely to be children of married couples (*p* < 0.002), reported lower perceived residential density (*p* < 0.001), higher perceived land use mix – diversity (*p* < 0.001), and lower levels of perceived crime (*p* = 0.003). The percent of adolescents from each SES/walkability stratum is as follows, 21.8% of adolescents lived in low SES, low walkable areas; 28.2% in low SES, high walkable areas; 26.7% in high SES, low walkable areas; and 23.3% in high SES, high walkable areas. A weak positive correlation was found between the subjective and objective indices of activity-friendliness (*r* = 0.11; *p* < .01). Detailed socio-demographic characteristics of the sample with valid GIS, survey, and accelerometer data are presented in Table 1.

**Table 1:** Sample characteristics (*N* = 524)

Characteristic	Statistic
Adolescent’s age, mean (SD)	15.78 (1.62)
Adolescent’s sex, *%* male	44.5
Adolescent’s race/ethnicity, %	
New Zealand European	69.9
Māori	3.1
Pacific Peoples	1.5
Asian	12.0
Other European	6.5
Other	6.5
Highest educational attainment in household, %	
Did not complete secondary education	2.4
Secondary – Completed school certificate	5.8
Secondary – completed sixth form certificate	5.1
Secondary – Completed University entrance or NCEA	7.1
Completed national or trade certificate	12.9
Completed and advanced certificate or diploma	11.2
Tertiary – Completed bachelor’s degree	20.8
Tertiary – Completed postgraduate study	22.6
Tertiary – completed doctorate degree	2.6
Marital status of parents/custodians, %	
Married	65.9
Widowed / divorced / separated	12.0
Single and never married	4.8
Living with partner	11.3
Place of residence (study site), %	
Auckland	78.4
Wellington	21.6
Adolescents from each stratum, %	
Low SES/ Low Walkable	21.8
Low SES/ High Walkable	28.2
High SES/ Low Walkable	26.7
High SES/ High Walkable	23.3
Length or residence at current address (years), mean (SD)	8.09 (7.07)
Length or residence in neighborhood (years), mean (SD)	10.92 (8.42)
Accelerometer wear time (valid days), mean (SD)	7.37 (0.86)
Accelerometer wear time (hr/day), mean (SD)	13.79 (1.38)
Moderate-to-vigorous physical activity (average min/day), mean (SD)	112.14 (36.95)
Sedentary time (average min/day), mean (SD)	354.80 (98.84)

N for some variables is reduced due to missing data. Accelerometer wear time (hr/day) = average number of valid hours per valid day. Sedentary time (average min/day) = average minutes of sedentary per valid day. Missing values: adolescent’s age (0%), adolescent’s sex (0%), adolescent’s race/ethnicity (0.6%), highest educational attainment in household (9.5%), marital status (6.0%), place of residence (study site) (0%); length of residence at current address and neighborhood (6.6%); accelerometer wear time variables, moderate-to-vigorous physical activity and sedentary time (0%)

### Descriptive statistics and socio-demographic correlates of objectively-assessed MVPA and sedentary time

The means and standard deviations of the perceived and objective environmental attributes are shown in Table 2, while Table 1 reports the average daily minutes of objectively-assessed MVPA and sedentary time. Participants met the PA guidelines by accumulating approximately 114 min/day of MVPA and on average accumulated ~354 min/day of sedentary time during accelerometer wear time (828 min/day). Participants from Wellington tended to accumulate, on average, 11% less MVPA than their Auckland counterparts.

**Table 2:** Descriptive statistics of perceived and objective environmental attributes (*N* = 524)

Environmental attribute	Th. range	M (SD)	Environmental attribute (cont.)	Th. range	M (SD)
Perceived (NEWS-Y)					
Residential density	1-750	115.0 (78.6)	Pedestrian/automobile traffic safety	1-4	2.96 (0.42)
Land use mix - diversity	1-5	2.72 (0.73)	Crime	1-4	1.38 (0.46)
Land use mix - access	1-4	3.30 (0.67)	Parking difficult in local shopping areas	1-4	2.19 (0.75)
Street connectivity	1-4	2.65 (0.55)	Physical barriers to walking	1-4	1.47 (0.74)
Walking facilities	1-4	2.99 (0.60)	Hilly streets	1-4	2.42 (0.89)
Aesthetics	1-4	2.79 (0.58)			
Composite subjective index of activity-friendliness	-∞, ∞	0.00 (1.99)	Composite subjective index of non-sedentariness	-∞, ∞	0.00 (2.89)
Objective (GIS)					
Net residential density (dwellings/km^2^)	0-∞		Street intersection density (intersections/km^2^)	0-∞	
250 m buffers		781.7 (560.4)	250 m buffers		154.5 (79.3)
500 m buffers		740.3 (513.8)	500 m buffers		147.5 (56.6)
1 km buffers		660.6 (459.6)	1 km buffers		146.2 (46.9)
2 km buffers		597.1 (409.1)	2 km buffers		143.9 (39.7)
Cul-de-sac density (cul-de-sacs/km^2^)	0-∞		Transit stops density (stops/km^2^)	0-∞	
250 m buffers		26.8 (30.0)	250 m buffers		34.1 (43.9)
500 m buffers		29.9 (27.7)	500 m buffers		36.0 (29.6)
1 km buffers		32.3 (24.4)	1 km buffers		36.0 (19.5)
2 km buffers		30.5 (10.7)	2 km buffers		33.4 (14.8)
Land use mix (entropy score)	0-1		Number of parks	0-∞	
250 m buffers		0.12 (0.14)	250 m buffers		1.13 (1.15)
500 m buffers		0.19 (0.18)	500 m buffers		2.40 (1.93)
1 km buffers		0.24 (0.19)	1 km buffers		6.85 (3.95)
2 km buffers		0.29 (0.21)	2 km buffers		22.63 (10.50)
Composite objective index of activity-friendliness (2 km buffer)		0.00 (1.71)	Composite objective index of non-sedentariness	-∞, ∞	NA

*NEWS-Y* Neighborhood Walkability Scale – Youth, *GIS* Geographic Information Systems, *Th.* theoretical. N for some variables is reduced due to missing data. Missing values: Residential density (0.2%), Land use mix – diversity (0%), Land use mix - access (0%), Street connectivity (1.7%), Walking facilities (1.1%), Aesthetics (0.6%), Pedestrian/automobile traffic safety (1.5%), Crime (1.7%), Parking difficult in local shopping areas (0.4%), No major barriers (0%), Hilly streets (0%), Composite subjective index of activity-friendliness (2.1%), Composite subjective index of non-sedentariness (3.4%), Composite objective index of activity-friendliness – 2 km buffer (0%). 0.6% of missing data on Net residential density, Street intersection density, Cul-de-sac density and Number of parks – 500 m and 1 km buffers. No missing data on the remaining GIS variables. Composite objective indices of non-sedentariness not computed as no objective environmental correlates of sedentary time were found

Perceived residential density, crime, and physical barriers to walking were low relative to their maximal theoretical values on the subscales, while perceived land use mix – access was high (Table 2). Higher average values on objective net residential and street intersection densities were observed for smaller buffer sizes. The opposite was true for objective land use mix, number of parks and cul-de-sac density (Table 2).

Adolescent’s sex and age were significant correlates of objectively-assessed MVPA and sedentary time. Specifically, girls accumulated 16.5% (95% CI: 11.7%, 21.0%; *p* < 0.001) less MVPA and 4.5% (95% CI: 0.4%, 8.8%; *p* = 0.032) more sedentary time than boys. Each one-year increase in age was associated with a 6.1% (95% CI: 4.4%, 7.9%; *p* < 0.001) decrease in MVPA and a 6.4% (95% CI: 5.0%, 7.8%; *p* < 0.001) increase in sedentary time. Participants from Wellington tended to accumulate, on average, 10.7% (95% CI: 1.0%, 20.2%; *p* = 0.015) less MVPA than their Auckland counterparts. No other significant socio-demographic correlates of adolescents’ objectively-assessed MVPA or sedentary time were found (all *p* > 0.286). Yet, these were included in the regression models of environmental correlates of sedentary time as they were a priori selected and the large sample size permitted their inclusion.

### Associations of perceived and objective environmental attributes with objectively-assessed MVPA

Three out of 11 perceived environmental correlates were significantly positively related to MVPA (Table 3). These included perceived land use mix – diversity, street connectivity, and aesthetics. For example, a unit higher score on perceived street connectivity was associated with a 6.3% (95% CI: 1.5%, 11.4%) higher average level of MVPA. Only two of 24 GIS-based environmental attributes were positively associated with MVPA. These were gross residential density and number of parks within 2 km from home. No support for curvilinear relationships between environmental attributes and MVPA was found.

**Table 3:** Associations of perceived and objective environmental attributes with objectively-measured moderate-to-vigorous physical activity (average daily minutes) (*N* = 522)

Environmental attribute	exp(b)	exp(95% CI)	*p*
Perceived (NEWS-Y)			
Residential density	1.000	1.000, 1.001	.907
Land use mix – diversity	**1.049**	**1.011, 1.088**	**.010**
Land use mix – access	1.039	0.999, 1.081	.056
Street connectivity	**1.063**	**1.015, 1.114**	**.010**
Walking facilities	0.975	0.934, 1.018	.242
Aesthetics	**1.052**	**1.005, 1.101**	**.030**
Pedestrian/automobile traffic safety	1.055	0.991, 1.123	.092
Crime	0.973	0.918, 1.031	.343
Parking difficult in local shopping areas	0.993	0.958, 1.028	.672
Physical barriers to walking	0.985	0.950, 1.021	.389
Hilly streets	1.000	0.970, 1.030	.982
Composite subjective index of activity-friendliness^a^	**1.025**	**1.012, 1.039**	**<.001**
Objective (GIS)			
Net residential density (100 dwellings/km^2^)			
250 m buffers	1.006	0.999, 1.013	.093
500 m buffers	1.000	0.993, 1.007	.976
1 km buffers	1.000	0.992, 1.008	.990
2 km buffers	**1.011**	**1.000, 1.022**	**.041**
Street intersection density (10 intersections/km^2^)			
250 m buffers	1.000	0.997, 1.004	.886
500 m buffers	1.002	0.997, 1.006	.448
1 kmbuffers	0.998	0.992, 1.004	.501
2 km buffers	1.002	0.995, 1.010	.491
Cul-de-sac density (10 cul-de-sacs/km^2^)			
250 m buffers	0.998	0.989, 1.007	.636
500 m buffers	0.993	0.983, 1.002	.121
1 kmbuffers	0.992	0.982, 1.003	.175
2 km buffers	0.984	0.956, 1.013	.274
Transit stop density (10 stops/km^2^)			
250 m buffers	1.004	0.997, 1.010	.242
500 m buffers	0.999	0.989, 1.008	.768
1 km buffers	0.999	0.983, 1.015	.869
2 km buffers	1.012	0.988, 1.036	.323
Number of parks (contained in or intersected by buffer)			
250 m buffers	0.995	0.973, 1.019	.698
500 m buffers	0.996	0.983, 1.010	.573
1 km buffers	0.999	0.992, 1.006	.770
2 km buffers	**1.003**	**1.000, 1.006**	**.024**
Land use mix			
250 m buffers	1.002	0.815, 1.233	.982
500 m buffers	0.989	0.839, 1.165	.891
1 km buffers	1.018	0.861, 1.203	.832
2 km buffers	1.067	0.875, 1.302	.515
Composite objective index of activity-friendliness^a^	**1.025**	**1.005, 1.046**	**.013**

exp(b) = antilogarithm of regression coefficient; exp.(95% CI) = antilogarithms of 95% confidence intervals; *p* = *p* value. Values of exp.(b) are to be interpreted as the proportional increase (or decrease) in objectively-assessed moderate-to-vigorous physical activity associated with a 1 unit increase of the environmental attribute. Values >1.00 indicate a positive associations (increase), while values <1.00 indicate a negative association (decrease). All regression coefficients were adjusted for adolescents’ age, sex, parental/custodian marital status, highest educational attainment in the household, length of residence in the neighborhood, administrative-unit socio-economic status, accelerometer wear time, and study site. Models accounted for administrative-unit and school clustering. Statistically significant (*p* < .05) environmental correlates are in bold

^a^sum of z-values of perceived or objective environmental attributes positively related to moderate-to-vigorous physical activity

Associations of perceived and objective environmental attributes with objectively-measured MVPA did not significantly differ by adolescent’s sex except for objective gross residential density within 2 km from home. Specifically, while no significant association was found in girls [exp(b): 0.999; 95% CI: 0.986, 1.012; *p* = 0.981], a positive association was found in boys [exp(b): 1.019; 95% CI: 1.007, 1.031; *p* = 0.002].

A composite subjective environmental index of activity-friendliness defined as the sum of the standardized values (z-scores) of perceived land use mix - diversity, street connectivity and aesthetics, was constructed. The index was linearly positively related to MVPA (Table 3). A 1 unit higher score on the index was associated with 2.5% (95% CI: 1.2%, 3.9%) more MVPA. The estimated difference in MVPA between those with the 1st quartile (−1.28) and 3rd quartile (1.39) observed values on the subjective index of activity-friendliness was 6.8% (higher MVPA in those with the highest score on the index), equivalent to approximately 8 min/day or 56 min/week. Adolescent’s sex did not moderate the association of the composite subjective environmental index of activity-friendliness with MVPA.

A composite objective environmental index of activity-friendliness, consisting of the sum of the z-scores of gross residential density and number of parks within 2 km distance from home, was also positively related to MVPA (Table 3), with associations being sex specific, i.e., significant in boys [exp(b): 1.040; 95% CI: 1.016, 1.066; *p* = 0.001] but not in girls [exp(b): 1.006; 95% CI: 0.980, 1.033; *p* = 0.641]. Overall, the estimated difference in MVPA between participants with the 1st quartile (−1.17) and 3rd quartile (1.15) observed values on the objective environmental index of activity-friendliness was 5.7%, corresponding to approximately 6.4 min of MVPA/day or 45 MVPA min/week.

A weak positive correlation was found between the subjective and objective indices of activity-friendliness (*r* = 0.11; *p* < .01). When these indices of activity-friendliness were entered in a main-effect model of MVPA, the objective index was no longer a significant correlate of MVPA [exp(b): 1.017; 95% CI: 0.996, 1.038; *p* = 0.106], while the subjective index showed only a slightly attenuated effect [exp(b): 1.022; 95% CI: 1.008, 1.036; *p* = 0.002]. In contrast, both indices were significantly correlated with MVPA in a model including sex as a moderator of the effect of the objective index. The regression coefficients of this model were exp.(b) = 1.022 (95% CI: 1.008, 1.036; *p* = 0.001) for the subjective index for the total sample (as sex was not a moderator for this index), exp.(b) = 0.998 (95% CI: 0.972, 1.025; *p* = 0.886) for the objective index in girls, and exp.(b) = 1.032 (95% CI: 1.008, 1.058; *p* = 0.010) for the objective index in boys.

### Associations of perceived and objective environmental attributes with objectively-assessed sedentary time

Five significant perceived environmental correlates of sedentary time were identified (Table 4). Perceived land use mix – diversity, street connectivity, aesthetics and pedestrian/automobile traffic safety – were negatively related to objectively-assessed sedentary time, while perceived physical barriers to walking (e.g. motorway, railway lines, rivers) were positively related to sedentary time. For example, a one unit higher score on the subscale of perceived pedestrian/automobile traffic safety was predictive of 4.8% (95% CI: 0.6%, 8.9%) fewer average daily minutes of sedentary time (Table 4). No significant objective environmental associates of sedentary time were found. Associations of perceived and objective environmental attributes with objectively-measured sedentary time did not significantly differ by adolescent’s sex. No support for curvilinearity in associations was found (all associations were linear).

**Table 4:** Associations of perceived and objective environmental attributes with objectively-measured sedentary time (average daily minutes) (*N* = 524)

Environmental attribute	exp(b)	exp(95% CI)	*p*
Perceived (NEWS-Y)			
Residential density	1.000	1.000, 1.001	.170
Land use mix – diversity	**0.968**	**0.944, 0.993**	**.014**
Land use mix – access	0.974	0.948, 1.002	.062
Street connectivity	**0.967**	**0.936, 0.999**	**.043**
Walking facilities	1.017	0.987, 1.048	.259
Aesthetics	**0.962**	**0.931, 0.993**	**.015**
Pedestrian/automobile traffic safety	**0.952**	**0.911, 0.994**	**.026**
Crime	1.032	0.991, 1.074	.128
Parking difficult in local shopping areas	1.019	0.994, 1.044	.129
Physical barriers to walking	**1.034**	**1.009, 1.060**	**.007**
Hilly streets	1.005	0.984, 1.027	.615
Composite subjective index of non-sedentariness^a^	**0.986**	**0.980, 0.993**	**<.001**
Objective (GIS)			
Gross residential density (100 dwellings/km^2^)			
250 m buffers	0.999	0.994, 1.004	.734
500 m buffers	1.001	0.996, 1.006	.771
1 km buffers	1.001	0.995, 1.006	.837
2 km buffers	0.996	0.989, 1.003	.281
Street intersection density (10 intersections/km^2^)			
250 m buffers	1.000	0.997, 1.002	.786
500 m buffers	0.999	0.996, 1.002	.487
1 km buffers	1.000	0.996, 1.004	.901
2 km buffers	0.981	0.933, 1.030	.428
Cul-de-sac density (10 cul-de-sacs/km^2^)			
250 m buffers	1.000	0.994, 1.006	.941
500 m buffers	1.003	0.997, 1.010	.305
1 km buffers	1.033	0.957, 1.116	.399
2 km buffers	1.015	0.994, 1.037	.146
Transit stop density (10 stops/km^2^)			
250 m buffers	0.999	0.994, 1.003	.587
500 m buffers	1.000	0.993, 1.007	.983
1 km buffers	1.004	0.993, 1.016	.440
2 km buffers	0.997	0.981, 1.014	.741
Number of parks (contained in or intersected by buffer)			
250 m buffers	1.008	0.992, 1.024	.334
500 m buffers	1.004	0.995, 1.014	.384
1 km buffers	1.003	0.998, 1.008	.216
2 km buffers	1.000	0.998, 1.002	.805
Land use mix			
250 m buffers	0.993	0.859, 1.147	.918
500 m buffers	1.000	0.892, 1.121	.998
1 km buffers	1.029	0.916, 1.156	.629
2 km buffers	0.925	0.806, 1.062	.262

exp(b) = antilogarithm of regression coefficient; exp.(95% CI) = antilogarithms of 95% confidence intervals; *p* = *p* value. Values of exp.(b) are to be interpreted as the proportional increase (or decrease) in objectively-assessed sedentary time associated with a 1 unit increase of the environmental attribute. Values >1.00 indicate a positive associations (increase), while values <1.00 indicate a negative association (decrease). All regression coefficients were adjusted for adolescents’ age, sex, parental/custodian marital status, highest educational attainment in the household, length of residence in the neighborhood, administrative-unit socio-economic status, accelerometer wear time, and study site. Models accounted for administrative-unit and school clustering. Statistically significant (*p* < .05) environmental correlates are in bold

^a^sum of z-values of perceived environmental attributes negatively related to sedentary time minus environmental attributes positively related to sedentary time

A composite subjective environmental index of non-sedentariness was computed, consisting of the sum of the standardized values (z-scores) of perceived land use mix - diversity, street connectivity, aesthetics, pedestrian/automobile traffic safety, minus the z-scores of perceived physical barriers to walking. The index was linearly negatively related to objectively-assessed sedentary time (Table 4). A 1 unit higher score on the index was associated with 1.4% (95% CI: 0.6%, 2.0%) fewer average daily minutes of sedentary time. The predicted difference in sedentary time between those with the 1st quartile (−1.83) and 3rd quartile (1.84) observed values on the subjective index of non-sedentariness was 5.7% (higher sedentary time in those with the lowest score on the non-sedentariness index), equivalent to ~20 min/day.

## Discussion

In this study we estimated the associations of GIS-determined and perceived walkability components in individual residential buffer zones with accelerometer-assessed MVPA and ST in NZ adolescents. Overall, most of the objectively-measured individual built environment features showed no significant association with the outcomes (two out of 24 showed significant association), and among those that did, it was only for the for the 2 km buffer. Also, only three of the 11 perceived environmental features were significantly associated to MVPA. There were no significant associations with ST and objective environment features. But there were five perceived environmental features that were significantly associated with ST. We explain further.

### MVPA and neighborhood environmental index of activity friendliness

The *objective* environmental index of activity friendliness consisting of the number of parks within 2 km from home, and net residential density and the *subjective* index of activity friendliness consisting of perceived land use mix – diversity, street connectivity, and aesthetics, were significantly and positively associated with MVPA in New Zealand adolescents. The estimated difference in MVPA between participants with the minimum and maximum observed values on the *objective* and *subjective* environmental index of activity-friendliness was approximately 6 and 8 min of MVPA/day (45 and 56 MVPA min/week) respectively. When both subjective and objective indices of activity-friendliness were entered in a main-effect model of MVPA, the objective index was no longer a significant correlate of MVPA, however, both indices were significant correlates of MVPA in a model including sex as a moderator of the objective index.

While higher average values of objective net residential and street intersection densities were observed for smaller buffer sizes, for objectively measured land use mix, number of parks and cul-de-sac density, the relationship was the opposite. Consistent with several other studies [48–57] we confirm previous research of a significant association between number of parks and MVPA in adolescents. In these studies, proximity to parks seemed to play an important role in the engagement of PA of adolescents even in unfavourable weather conditions [48]. Parks are spaces where adolescents have the opportunity to meet and play sport or ride a bicycle, or skateboard away from traffic. Parks are also the places where other recreational facilities can be found. When in close proximity to the home they are also destinations that can be actively travelled to. Indeed, it has been reported [54] that nearby neighborhood and major parks were significantly related to active sports and wheel-based PA, particularly in adolescent girls [54].

The positive association between net residential density with MVPA is consistent with some adolescent studies [51, 56, 58] but not others [59]. The latter study reported that adolescents residing in Belgium were likely to be more accustomed to engaging in PA (cycling) irrespective of low neighborhood walkability (low connectivity and low residential density). High residential density is indicative of access to a variety of destinations (friends’ homes, or transit stops) and improved infrastructure. This feature has also been found to be frequently associated with adult PA [60, 61].

While mixed land use is often reported as one of the most robust correlates for adolescents [62], we only found a significant association with perceived land use mix diversity and MVPA. Others have shown that awareness of a variety of services, shops and retail encouraged MVPA in adolescents, and having a place to go within walking distance from school [63] indicative of diverse mixture of destinations, was predictive of PA in adolescents. Perceived mixed land use is also an often reported correlate of adult PA [64]. In our study and similar to previous research [63], we showed the strongest associations between MVPA and perceptions of land use mix diversity (*p* = 0.010), followed by land use mix access (*p* = 0.056).

The appearance of the perceived natural and physical environment seems to play a role in objectively-assessed PA in New Zealand adolescents. An aesthetically pleasing environment translates into neighborhoods with trees in the streets, greenery present, interesting or beautiful natural things to look at, and attractive buildings [36]. However, most studies [65, 66] have observed an association between perceived aesthetics and self-reported PA but not with objectively assessed PA [62, 67]. Unlike others we have found aesthetics to be an important environmental feature for adolescents. Other New Zealand research with 9–11 year old children identified the most supportive settings for children’s mobility were older, more established neighbourhoods, rather than greenfield developments [68], and these may be more aesthetically pleasing.

Street connectivity is a measure of how the road network is configured. A ‘well-connected’ street network tends to have blocks of shorter lengths, more crossing points (either controlled or uncontrolled), and more direct links between origins and destinations. As street connectivity decreases, block lengths become longer, the network becomes less permeable (including cul-de-sacs), and network travel distances become longer between origin and destination [21]. Perceived street connectivity, the number of intersections in a particular area, was also positively associated with MVPA in adolescents in this study. Most studies examining this particular variable reported contradicting results [66, 69, 70]. The one study that has investigated both perceived and objective measures of the built environment with objectively assessed PA did not report any association with street connectivity [15] but found only the sidewalk characteristics were associated with light intensity PA and sedentary behavior [15]. Previous studies noted that greater street connectivity may elicit greater safety concerns regarding traffic among younger children [71]. However, for this adolescent population, it may be they are able to negotiate the traffic risk, to receive the benefits (e.g. shorter commute distance, opportunity for route variation) of travelling through a better connected street network [72]. To support this argument, there is a substantial body of research demonstrating that populations, especially when segmented by age, respond differently in terms of physical activity and sedentary time, when exposed to different built environments (see [73] for examples).

Both composite subjective and objective indices of activity friendliness were linearly and positively associated with MVPA confirming the importance of the identified subjective and objective environmental variables with PA. When entering both subjective and objective indices of activity-friendliness in a main-effects model of MVPA, only the subjective index remained significant indicating that the perceived environment has greater explanatory power compared to the objective environment for the overall sample. This result may indicate that perceived data should be considered to inform structural interventions. When sex was entered as the moderator of the objective index, the subjective index was significant for both girls and boys (i.e., the total sample) and the objective index only for boys. These findings indicate that girls’ perception of the built environment has a greater effect on their MVPA than the objective environment. It is possible that girls’ participation in PA is influenced by their perception about the environment (aesthetics/safety and street connectivity) while in boys it is influenced by the actual environment for example presence of parks and high residential density.

### Sedentary behavior and neighbourhood environment index of non-sedentariness

There were no significant associations between the objective environmental measures assessed in this study and sedentary time and. However, a 2011 review showed associations with topography and neighbourhood type (suburban versus urban) with youth sedentary behavior [74]. It could be that different objective environment measures are associated with MVPA and sedentary behavior and this requires further investigation. In agreement with the review [74], our study showed five perceived environmental features were significantly negatively associated with sedentary time, namely perceived land use mix – diversity, street connectivity, aesthetics, and pedestrian/automobile traffic safety. The predicted difference in sedentary time between those with the minimum and maximum observed values on the *subjective index of non-sedentariness* was equivalent to 20 min/day. Perceived physical barriers to walking were positively related to sedentary time. There was a sex effect where girls accumulated more sedentary time than boys, and a city effect whereby adolescents from Wellington tended to accumulate less MVPA than their Auckland peers.

Few studies have examined the associations between the built environment and ST in adolescents (and children and adults), and findings to date are equivocal [53, 75, 76]. Only two studies in adolescents have reported significant associations – one between the presence of hills in the neighborhood and self-reported ST in adolescent girls [75], and the other for sidewalk characteristics and objectively measured sedentary time in US male adolescents [53]. In children, the number of cars available in the household and distance between home and school [77] were positively associated with the likelihood of being driven to school [78]. Of the 17 studies investigating the associations between neighborhood environmental attributes and ST in adults [76], only two studies reported associations between objective total ST and the neighborhood environment [79, 80]. Kozo and colleagues [80] collected information from approximately 2000 adults living in US neighborhoods of high or low walkability. Even though a non-significant association was observed between total sedentary time and neighborhood walkability, the trend supported their hypothesis that total ST was associated with low-walkable neighborhoods. They discussed that attempting to observe associations with total sitting time and neighborhood walkability may have not been completely appropriate as most of the sitting during the day takes place in the work environment, rather than the wider built environment. Similarly in our study, time spent at school contributed substantially to ST accumulation. An additional perceived environmental feature that was significantly and positively associated with less sedentary time was perceived pedestrian/automobile traffic safety. The safer pedestrian and automobile traffic was perceived the less sedentary time was accumulated. Adolescents’ perceived physical barriers to walking (e.g., the presence of large infrastructural or natural barriers such as freeways, railway lines, and rivers) were significantly and positively related to sedentary time. Such perceived barriers may have triggered the need to travel by car and/or remain indoors engaging in screen time, as it is too difficult to access desired destinations.

Unlike the effect of sex as a moderator in the combined assessment of the contribution of subjective and objective indices of activity-friendliness to the explanation of adolescents’ MVPA, associations of perceived and objective environmental attributes with objectively-measured sedentary time did not significantly differ by adolescent’s sex.

In agreement with previous research girls accumulated more minutes in sedentary time compared to boys. Matthews and colleagues [81] found that among 6326 US participants, females were more sedentary than males before the age of 30 years. Similarly, the prevalence of >2 h per day of screen time was higher in female Saudi and British adolescents compared with males [82]. However, in Australian [83] and Finnish [84] adolescents, it was observed that females were less likely to engage in small screen recreation time or sedentary activities. Apart from Matthews [81] the studies mentioned above measured ST via self-report, and as discussed earlier, it may be that different population groups respond differently to environmental cues [73].

The strengths of this study include the perceived and objective measurement of the built environment with the objective measurement of PA and ST. Length of residence in the neighborhood was introduced as a covariate to capture neighborhood familiarity for the first time. To explain PA and ST of New Zealand adolescents, we provided a combined assessment of the contribution of subjective and objective indices of activity-friendliness. Our findings point to a 2 km buffer as being appropriate to test for these associations in this population. However, using this larger buffer, rather than a smaller one, say 250 m, will likely smooth the environmental variability around a specific school, while potentially increasing the between school variability. Therefore, the larger buffer may be capturing ‘unmeasured’ neighbourhood attributes that are not related to the built environment per se, and can not necessarily be controlled for in the modelling approaches. It was clear that sex plays an important role. There are limitations to the study. Cross-sectional studies cannot infer causation but can only highlight associations for further investigation. In line with similar research [85], our response rate was low, limiting generalisability to other population groups. Overall, the sample were highly active, accumulating an average of 112 min of MVPA per day. It is possible that self-selection bias existed in this study, with more active youth enrolling with the study, and less active youth choosing not to participate. Future strategies to improve response rates, improve generalisability, and reduce exclusion due to insufficient data are warranted [86–88]. Measuring domain specific instead of total ST may have enhanced the accuracy of ST captured. Our study highlights two issues that need to be explored further in future research: 1) ensuring sensitivity in deriving the dependent variable (e.g., total sedentary time versus neighborhood-specific sedentary time), and 2) the assessment of domain-specific ST (e.g., screen time versus passive transport).

## Conclusions

Relationships between the built environment and objectively-assessed MVPA and ST in a large group of New Zealand adolescents were observed and were in the expected directions. The combined assessment of the contribution of subjective and objective indices of activity-friendliness to the explanation of NZ adolescents’ PA and ST showed positive relationships with MVPA for the subjective index only, indicating that the subjective environment has a greater explanatory power. When sex was entered in the model as a moderator of the objective index, the subjective index was significant for both girls and boys and the objective index only for boys. For ST, associations of perceived and objective environmental attributes with objectively-measured sedentary time did not significantly differ by adolescent’s sex. Further research is warranted to understand the relationships of ST with the built environment.

## Appendix 1

**Table 5:** GIS measures of the built environment

Measure	Calculation
Gross residential density	Dividing number of residential dwellings obtained from the 2013 New Zealand census by the area of the buffer
Street intersection density	Dividing the number of 3 or more-way intersections derived from the walkable road network by the area of the buffer
Cul-de-sac density	Dividing the culs-de-sac derived from the walkable road network by the area of the buffer
Transit stop density	Number of transit stops (bus, train, and ferry in Auckland, and bus and train in Wellington) by the area of the buffer
Number of parks	Contained in or intersected by the buffer were calculated using park data source from territorial authorities
Land use mix^a^	Land use mix for each buffer was calculated using an entropy formula [89]

^a^The parcel level land use data used to calculate land use mix was compiled from a variety of sources: territorial authority zoning data, territorial authority points of interest data sourced in 2013/2014 (e.g., parks, libraries), and Zenbu online business directory data extracted in 2014

## Appendix 2

**Table 6:** Neighborhood Environment Walkability Scale – Youth (NEWS-Y)

Subscales	Calculation
Residential density	Weighted sum of items reflecting perceived density of housing, ranging from predominantly single-family dwellings to high-rise buildings with more than 20 stories [31]
Land use mix – diversity	Average perceived walking proximity (i.e., average of five-point ratings ranging from ≤5 min walk to ≥30 min walk) from home to 26 types of destinations (e.g., supermarket, library, post office, any school, the participant’s school, bus or train stop, park, gym or fitness facility, etc.)
Remaining nine scales	Average ratings of items answered on a four-point Likert scale (1 = strongly disagree to 4 = strongly agree)
